# Impact of dengue fever on depression, anxiety, and stress symptoms in Esmeraldas Province, Ecuador: a prospective cohort study

**DOI:** 10.1186/s41182-024-00625-0

**Published:** 2024-09-27

**Authors:** Julio P. Salazar Buenaño, Fabián A. Zurita Alvarado, Ines Weyand, Tamara Rosero Montezuma, Boris Tapia, Cecilia Solis Olive, Karen Rosero, Pablo Bermudez, Federico Gobbi, Emmanuel Bottieau, Ralph Huits

**Affiliations:** 1 Centro de Investigaciones Para la Salud en América Latina, Pontificia Universidad Católica del Ecuador, Av. 12 de Octubre 1076, Apartado: 17-01-218, Quito, Ecuador; 2 Facultad de Medicina, Pontificia Universidad Católica del Ecuador, Av. 12 de Octubre 1076, Apartado: 17-01-2184, Quito, Ecuador; 3 Institute of Psychology, Goethe-Universität, Campus Westend, PEG Theodor-W-Adorno-Platz 6, 60323 Frankfurt am Main, Germany; 4 Posgrado de Medicina Familiar y Comunitaria, Pontificia Universidad Católica del Ecuador, Av. 12 de Octubre 1076, Apartado: 17-01-2184, Quito, Ecuador; 5 Department of Infectious Tropical Diseases and Microbiology, IRCCS Sacro Cuore Don Calabria Hospital, Via don A. Sempreboni, 5, 37024 Verona, Negrar Italy; 6 Department of Clinical Sciences, Institute of Tropical Medicine, 155 Nationalestraat, 2000 Antwerp, Belgium

**Keywords:** Dengue, Depression, Anxiety, Neurocognitive

## Abstract

**Background:**

Physical symptoms of dengue have been documented extensively, but knowledge gaps on dengue-associated mental health hazards remain. We investigated the frequency of psychiatric symptoms (depression, anxiety, and stress) and neurocognitive performance during the first year after a dengue episode.

**Methods:**

Using DASS-21 scores at 3, 6, and 12 months, we assessed depression, anxiety, and stress in anti-dengue IgM-positive adults and matched controls during the 2021 dengue season in Esmeraldas Province, Ecuador. Patients with DASS-21 scores ≤ 4 were considered normal; those with scores of 5–7, 8–10, and ≥ 11 indicated mild, moderate, and severe depression, respectively; cutoff scores for anxiety and stress were ≥ 5 and ≥ 9, respectively. We also assessed ‘delayed matching to sample’ (DMS) and ‘spatial working memory’ (SWM) using the Cambridge Neuropsychological Test Automated Battery.

**Results:**

We enrolled 102 cases and 78 controls. At 3 months, 90 cases and 70 controls were available for follow-up, among these 40/90 (44.4%) cases and 12/70 (17.1%) controls had DASS-21 scores ≥ 5 (RR 2.7, 95% CI [1.5–4.7]). Dengue remained a predictor for depression after adjusting for age, sex, and COVID-19 status. We observed no difference in anxiety between the groups, but stress scores increased at month 3 (RR 1.87, 95% CI [1.01–3.4]). DASS-21 scores normalized during follow-up. DMS and SWM did not differ between groups at 3 and 6 months. At month 12, cases had lower SWM than controls did (*p* value < 0.001).

**Conclusions:**

Care providers should be aware of dengue-associated mood disorders and facilitate timely referral to mental health services. Future longitudinal studies are warranted to validate our observations regarding the impact of dengue on mental health and neurocognitive status in affected patients.

**Supplementary Information:**

The online version contains supplementary material available at 10.1186/s41182-024-00625-0.

## Introduction

Dengue is a vector-borne disease caused by dengue virus (DENV). It is endemic in tropical countries and has emerged as a serious public health problem as the annual number of dengue cases worldwide increased from 23 million in 1990 to 104 million in 2017 [1]. The Pan American Health Organization reported 4.2 million new cases in the Americas in 2023, and in 2024, up to April, 3.4 million cases have been confirmed in this region [2]. Esmeraldas is a dengue endemic region in northwestern Ecuador; in 2023, a total of 1742 cases were reported, with a rate of 314.1/100 000 inhabitants [3]. Infection with any of the four DENV serotypes can lead to disease, ranging from a mild febrile illness to life-threatening conditions. Although the physical consequences of dengue are well documented, mental health hazards such as neurocognitive impairment, depression, anxiety, or stress associated with this infection have been characterized less frequently.

Sickness behavior is an acute syndrome described during infectious diseases, with reduced appetite, fatigue, sleep changes, and social withdrawal, symptoms that are shared with depression and anxiety [4]. An important difference is that sickness behavior is a phenomenon that exists for the duration of the acute illness, while depression and anxiety tend to be chronic and episodic, and minimum time stipulated to diagnose a major depressive disorder (according to DSM-V code F32-F33) is 2 weeks [5].

Sickness behavior, depression, and anxiety also share similar pathophysiological pathways. Increased cytokine levels during acute inflammation can activate the hypothalamic‒pituitary‒adrenal axis leading to an increase in cortisol levels, which can initiate neurotoxicity in neuronal networks related to mood [6].

Various viral infections have been related to neurologic symptoms [7]. In dengue, the frequency of these symptoms ranges from 4 to 21% [8]. Some authors have hypothesized that central nervous system involvement in *Flavivirus* infections leads to acute or long-term neurocognitive impairment [9].

Several studies have investigated the occurrence of depression related to acute dengue episodes. Hashmi et al. observed that a significant proportion of dengue patients—about 62.5%—experienced depression during their hospital stay in Pakistan [10]. Meanwhile, a retrospective case–control study by Gunathilaka et al. in Sri Lanka found that 15.1% of patients who had dengue 6 to 12 months prior were diagnosed with depression, compared to 7.5% in matched controls, though this difference was not statistically significant [11]. These studies do not include enough follow-up, at least 1 year, considering that 80% of major depressive disorder recoveries begin within the first year [12]. In addition, they do not account for sociodemographic factors related to mood and neurocognitive impairment; for instance, female sex has been identified as a risk factor for developing mood disorders [13], and neurocognitive impairment shows an increasing trend with age.

We conducted a prospective cohort study with the aim of investigating a putative association between dengue and symptoms of depression, anxiety, and stress, as well as between dengue and selected neurocognitive changes during a 12-month follow-up period.

## Methods

We designed a prospective study to assess the development of depression, anxiety, and stress symptoms as well as neurocognitive sequelae (attention, nonverbal memory, working memory, and executive function) in patients after an acute dengue episode compared to controls in Esmeraldas Province in Ecuador, South America, during the 2021 dengue season. The sample size was calculated based on an anticipated 20% prevalence of depression in the exposed group. as informed by previous studies published in the literature [10, 11], resulting in a sample size of 88 exposed and 88 non-exposed individuals, with significance level of 0.05 and power of 0.80. Expecting a 20% dropout rate, refusal to participate, and incomplete data, we invited 120 subjects to participate in each group, of which only 102 cases and 78 controls provided their consent.

Cases were consecutively selected from among patients who presented with compatible clinical features of dengue based on the WHO criteria (fever and two of the following: nausea, rash, arthralgia, or leucopenia) [14] at the emergency room or outpatient departments in San Rafael and La Concordia Health facilities, during the first week after symptom onset, the inclusion criteria were (a) IgM-positive (Wondfo Dengue IgG/IgM Antibody Test, Guangzhou Wondfo Biotech Co., Ltd., China) and (b) 18 to 65 years old, informed consent was obtained from the patients for follow-up per study protocol, starting at month 3, to avoid misdiagnosis with sickness behavior.

Age- and sex-matched controls were recruited from otherwise healthy individuals attending family planning and dentistry services at the same facilities and from the community. The controls were tested for anti-dengue immunoglobulin (Ig)G and IgM negativity at enrollment, 3 months before the mental health assessments.

Exclusion criteria were a history of psychiatric illness, clinical signs of neurological or rheumatic disease, pregnancy or substance abuse other than alcohol and failure to provide informed consent.

We used a structured questionnaire (see file S1) to obtain demographic, socioeconomic, and clinical data, including education level, household income, employment status, housing, medical and psychiatric history, and adverse life events. During each visit, we assessed the occurrence of inter-current febrile illness, dengue diagnosis, or COVID-19.

During the first and follow-up study visits at 3, 6, and 12 months, the Depression, Anxiety, Stress Scale 21 (DASS-21) was used to assess symptoms of depression, anxiety, and stress (see S2 file) [15, 16]. A validated Spanish language version of the DASS-21 scale [17] was administered to all participants by qualified and trained family physicians. This tool consists of 21 questions, 7 that assess anxiety [2, 4, 7, 9, 15, 19, 20], 7 for depression [3, 5, 10, 13, 16, 17, 21] and 7 for assessing stress [1, 6, 8, 11, 12, 14, 18]. The identity of the participants was encoded, and the results of the questionnaires and DASS-21 scores were recorded via smartphones in offline mode during home visits for later synchronization or stored and encrypted directly during programmed office visits using the digital data collection tool KoBoToolbox (http://www.kobotoolbox.org). During the study visits at 3, 6, and 12 months, we assessed the neurocognitive key domains attention, nonverbal memory, working memory and executive function that are relevant to mood disorders using the Cambridge Neuropsychological Test Automated Battery (see S3 file) (CANTAB®). CANTAB is a language-independent, culturally neutral, noninvasive assessment. We tested memory-controlling deficits of attention using Delayed Matched to Sample (DMS) testing and working memory, i.e., the storage of information for short periods of time to inform decision-making, using Spatial Working Memory (SWM) testing [18].

Means, ranges, and proportions were calculated for descriptive statistics; Chi-squared was used to test differences in baseline characteristics, two by two tables were constructed to calculate risk ratios for depression, anxiety, and stress. Crude odds ratios (ORs) with 95% CI were calculated to identify risk factors in univariate logistic regression models. When significant at the 10% level, a multivariate regression model was fitted to adjust for confounding factors and multiple predictors. The final model, which included COVID-19 and sex, was selected using stepwise backward elimination with the likelihood ratio test as the comparison test. After a normality test (Shapiro–Wilks), the Mann‒Whitney U test was used to determine differences in the medians of neurocognitive assessments between cases and controls, a subgroup analysis was performed on the neurocognitive scores that demonstrated significant differences.

Ethical approval was obtained from the Institutional Review Board at the Institute of Tropical Medicine Antwerp, Belgium, and the Ethics Committee of Pontificia Universidad Católica del Ecuador (MB-04-2021). All participants provided written informed consent. All participants who had a positive score on the DASS-21 scale were referred to the mental health service in the health center.

### Patient and public involvement

All the participants signed informed consent forms, and their results were communicated in each test. After the publication of this research, a public meeting in the community will be done to inform and discuss the results with the community.

## Results

### Demographics and baseline characteristics

**Fig. 1:**
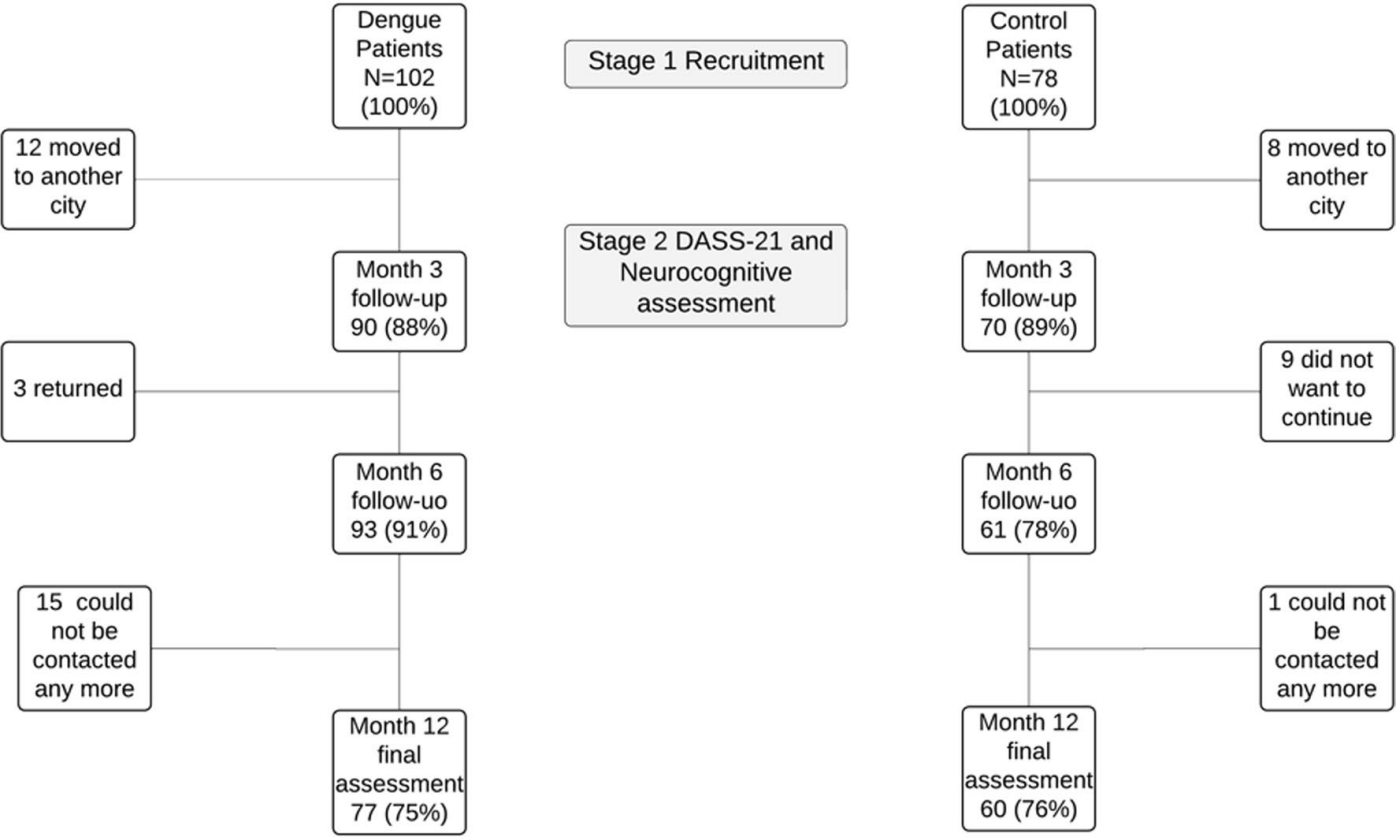
Participant flowchart

A total of 102 cases and 78 controls were recruited. Ninety cases (88.2%) and seventy controls (89.7%) were retained at the first visit (month 3), and seventy-seven cases (75.4%) and sixty controls (76.9%) completed the 12-month follow-up (Fig. 1). During follow-up, none of the controls contracted dengue, but two of them had febrile illness without an established etiology.

Cases and controls had similar ages (mean 35.5 years (range 18–65) and 37.6 years (range 18–65 years), and sex distributions, with 57% and 53% (*p* = 0.3) of female patients, respectively. Housing conditions were also comparable. However, the controls had higher educational levels and family incomes, and a higher proportion reported stressful life events and comorbidities. At the time of enrollment, the proportions of cases and controls who had COVID-19 diagnosed by PCR were comparable (22% vs. 34%, *p* = 0.09) (Table 1).

**Table 1:** Demographic and baseline characteristics

Variable	Cases (*n* = 102)	(%)	Controls (*n* = 78)	(%)	*p* value
Age					
18–39 years	72	(71)	48	(62)	0.2
40–55 years	25	(24)	21	(26)	0.2
>55 years	5	(5)	9	(12)	0.2
Gender					
Female	58	(57)	42	(53)	0.7
Male	44	(43)	36	(47)	0.7
Ethnic group					
African	18	(18)	20	(25)	0.3
Mixed race	79	(78)	56	(72)	0.3
Other	5	(4)	2	(3)	0.3
Level of education					
Primary	18	(18)	9	(12)	0.01*
High school	60	(59)	30	(38)	0.01*
University	24	(23)	39	(50)	0.01*
Housing					
Cemented	84	(82)	68	(87)	0.7
Adobe	4	(4)	0	(0)	–
Mixed	14	(14)	10	(13)	0.7
History of COVID-19	23	(22)	25	(34)	0.15
History of Chikungunya infection	10	(10)	10	(12)	0.5
Stressful life event	11	(11)	22	(28)	0.002*
Family history of depression	2	(2)	1	(1)	0.7
Family income					
< 425 USD	64	(64)	26	(33)	< 0.01*
425 USD	16	(16)	25	(33)	< 0.01*
> 425 USD	22	(20)	27	(34)	< 0.01*
Comorbidities	3	(3)	10	(7)	0.01*

### DASS-21 scores

At 3 months, 40/90 (44.4%) of cases and 12/70 (17.1%) of controls had DASS-21 depression scores ≥ 5 (RR 2.7, 95% CI [1.5–4.7]). Using a multivariate regression model that included age, sex, and COVID-19 status, we identified acute dengue episode as an independent risk factor for depression scores > 5 at 3-month post-infection (OR 4.9, 95% CI [2.3–11.8]).

We analyzed the COVID-19 and non-COVID-19 subgroups separately, after which the dengue group remained with higher DASS-21 depression scores (OR 5.7, 95% confidence interval [2.22–18]).

**Fig. 2:**
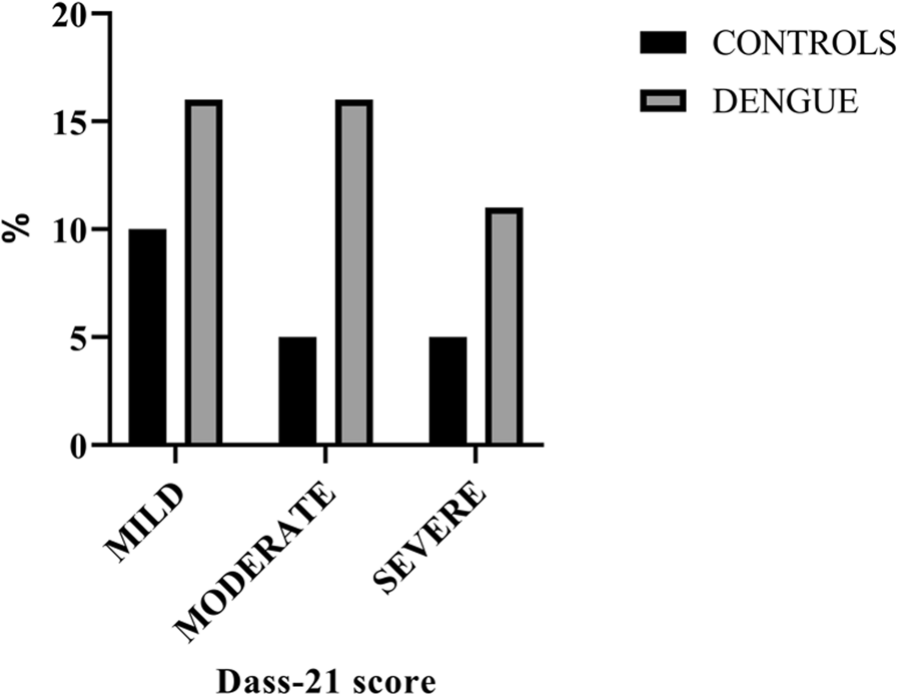
Depression scores at month 3

DASS-21 depression scores at month 3 showed that 16% of cases and 10% of the controls had mild depression scores (*p* = 0.06), 16% of cases and 4 % of the controls had moderate depression scores (*p* = 0.01), and 11% of cases and 4% of the controls had severe depression scores (*p* = 0.08) (Table 2 and Fig. 2).

**Table 2:** Depression scores at month 3

Depression scores	Cases (90)	%	Controls (70)	%	RR	C.I.
Overall	40	44	12	17	2.7	1.5–4.7
Mild	15	16	7	10	1.66	0.7–3.8
Moderate	15	16	3	4	3.88	1.1–12.9
Severe	10	11	2	4	3.88	0.8–17.1

During follow-up, the DASS-21 depression scores normalized, with no differences between groups (RR 0.92, 95% CI [0.43–1.9] and RR 0.25, 95% CI [0.5–1.24] at 6 and 12 months, respectively).

At months 3, 6, and 12, anxiety scores were normal, and there were no differences between groups (RR 1.69, 95% CI [0.77–3.7]; RR 0.76, 95% CI [0.3–1.9]; and RR 0.12, 95% CI [0.01–1.05]). Cases more frequently had stress scores ≥ 8 than did controls at month 3 (RR 1.87, 95% CI [1.01–3.4]), but the difference normalized at month 6 (RR 0.76, 95% CI [0.3–1.9]). Remarkably, at month 12, cases had stress scores ≥ 8 less frequently than controls did (RR 0.09, 95% CI [0.01–0.5]).

### Neurocognitive assessments

Spatial Working Memory is essential for informing decision-making, SWM testing assesses this executive function by measuring planned strategy, where low scores in selecting tokens among simultaneously presented squares of different colors (SWMS) indicate high strategy use. It also records working memory errors (SWME), i.e., the number of times the subject incorrectly revisits a colored square. Neither of the assessments differed between the groups at 3 and 6 months (see S4).

Visual memory and attention were tested using Delayed Matched to Sample test (DMS). The subjects are shown complex visual patterns, which they must select from a panel of four similar patterns that are shown after a brief delay. The outcome measures included the subject’s time to respond (latency), the number of correct patterns selected, and the probability of an error after a correct or incorrect response. At 3 months, the DMS measurements did not differ between the groups. At 12 months, no significant differences were observed in the DMS or SWM measurements between cases and controls (see S4).

**Fig. 3:**
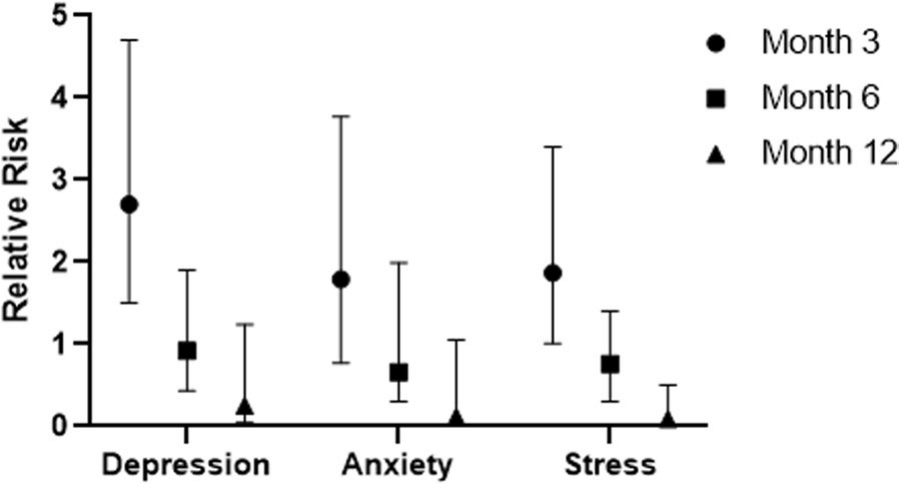
Relative risk (RR) of higher depression, anxiety, and stress scores cases compared to controls at months 3, 6, and 12

**Fig. 4:**
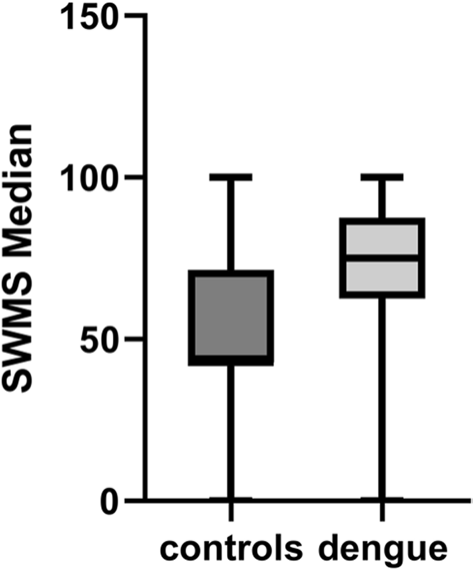
Strategy test (SWMS) after 12-month follow-up

However, 1 year after dengue, the planned strategy (SWMS) median scores for cases,75, was significantly worse than for controls, with a median of 42.86 (W = 1019, 95% CI, p value ≤ 0.001) (Figs. 3 and 4).

## Discussion

We assessed psychiatric symptoms and selected neurocognitive outcomes among persons who had IgM-positive dengue compared with healthy controls during the 2021 outbreak in Esmeraldas, Ecuador.

Using the DASS-21 scale, people who had dengue 3 months prior had higher depression and stress scores, but not anxiety scores, than healthy controls. In our study, the depression and stress scores normalized by months 6 and 12. Our findings contrast with those of Gunathilaka et al. [11] where high DASS-21 scores were observed in patients who were enrolled more than 1 year after a dengue episode. This can be explained because of the natural course of depressive disorders; normally, a depressive episode lasts 20 weeks, and patients can experience relapse for many years, with shorter and milder episodes, [19] which is why a long period of follow-up beyond 1 year is desirable in future research.

In our study, among outpatients, the highest depression and stress scores were observed during the first assessment after dengue episode. A cross-sectional study from Pakistan revealed elevated scores on the Hospital Anxiety and Depression Scale (HADS) in 60% of acute dengue inpatients, with a positive correlation between HAD scores and symptom severity [10]. Differences in design aside, lower depression scores in our study could have been caused by later capture of the first assessment of our patients, i.e., after the acute phase of the disease.

There is emerging evidence that COVID-19 can also be followed by depression and anxiety symptoms [20]. After logistic regression, we did not identify COVID-19 as a confounding factor for the observed associations between dengue and psychiatric symptoms; additionally, we performed a subgroup analysis (COVID-19 and no COVID-19), after which dengue remained as an independent risk factor for higher DASS-21 scores.

Acute and chronic viral and bacterial infections have been associated with neurocognitive impairment [21]; neurocognitive long-term sequelae, memory impairment, and language and learning difficulties have been documented after arboviral infections, such as Zika, chikungunya, Japanese encephalitis, and West Nile virus infections [22].

Significant impairment in planned strategy testing (SWMS) was detected in dengue cases, while visual memory was preserved (DMS). SWMS, which has an anatomical base in the prefrontal cortex, is a more demanding cognitive task [23]; instead, visual memory is more dependent on visual processing areas such as the occipital lobes [18]. Evidence is emerging that neuroinflammation induces neurocognitive impairment by disrupting neural networks in the prefrontal cortex [24], which can explain our findings. Using the CANTAB assessment tool, Sejvar et al*.* investigated neurocognitive outcomes 1.5 years after West Nile virus illness in the USA and concluded that neurocognitive performance did not differ between cases and controls [25]. A study in Mexico in dengue and Zika patients showed that transient neurocognitive impairment occurred, starting from 3 to 28 days after the acute episode, and it improved, though not completely, at 180 days [9]. The observed trend in this study contrasts with our findings, which show increasing impairment of domains over time. Future studies with longer follow-up periods are needed to fully understand the implications of declining executive functions in cases, over the 12 months following dengue.

A nationwide longitudinal study in Taiwan [26] showed a distinctly increased risk for the development of dementia in dengue patients.

The described sociodemographic factors related to depression are age, sex, educational level and income level, among others [27], and vary substantially across countries [28]. Our dengue and control patients differed in education and income levels, to evaluate possible confounding factors, we included these in several logistic regression models. Finally, the best model did not include these sociodemographic factors because they did not change the direction of the relationship between dengue and depression. Our groups also differed in recent stressful life events, which could be related to adjustment disorders [29]; however, these events occurred more frequently among the controls.

Several limitations apply to our study. We determined our sample size based on a prevalence estimate of 20% for depressive disorder. This decision was guided by local studies which report depression prevalence ranging from 8.7% to 35.4% [30, 31], depending on the population and region studied. In addition, international studies on depression in dengue patients reports prevalence rates ranging from 15 to 65% [10, 11]. However, this estimate may not precisely capture the specific prevalence within our study area, and the generalizability of our findings to other regions should be interpreted with caution.

We used IgM antibody testing to select dengue cases, potentially enrolling patients who were potentially infected with other *Flaviviruses* because of extensive serologic cross-reactivity in this genus. However, no other Flaviviruses have been reported to circulate in Ecuador for 5 (yellow fever virus) or 3 years (Zika virus).

Other viral emerging diseases (e.g., chikungunya) have been associated with the occurrence of symptoms of depression and anxiety [32]; however, no chikungunya cases have been reported in our population since 2018.

Although we systematically assessed the occurrence of febrile illness during follow-up visits, it is possible that asymptomatic dengue infections among controls were undetected.

Our study used screening tools for depression, anxiety, and stress. A psychiatric evaluation would be preferred to establish a diagnosis of mood disorders or anxiety in future research.

Impulsivity (a predisposition to action without adequate thought that is related to several psychological disorders, including mood disorders) has been associated with depression (OR 1.95; 1.28–2.97) [33], and it can explain the shorter delay in the dengue group in DMS test; in this sense, impulsivity assessment is a limiting aspect that should be taken into account in future research.

Finally, we only evaluated neurocognitive performance in a few key domains, with a follow-up that was limited to 12 months. Our observations on the progressive decline in Spatial Working Memory warrant longer follow-up, and additional key domains (social domains and response inhibition) should be assessed to comprehensively study the occurrence of neurocognitive deficits after dengue.

Our observations of higher DASS-21 scores in dengue patients compared to controls at 3 months should alert care providers to the increased risk of depression and stress after acute dengue and should facilitate timely referral to mental health services if necessary. Future studies with adequate follow-up are warranted to validate our observations regarding the impact of dengue on mental health and neurocognitive status in affected patients and communities.

## What is already known about this topic

Previous studies have shown that depression and anxiety can present during acute dengue episodes, but there are few long-term data and little information about stress. Research on neurocognitive performance in dengue patients is scarce and has not been evaluated through automated or prospective assessments.

## What this study adds

Dengue patients had higher depression and stress scores after 3 months of dengue episode and this scores normalized by months 6 and 12. Neurocognitive impairment in working memory is present 1 year after dengue.

## How this study might affect research, practice, or policy

Health care workers should be aware of the increased risk of depression after acute dengue.

## Supplementary Information

Supplementary Material 1.

## Data Availability

Materials are added as supporting information for this submission, and dataset used in the current study will be available from the corresponding author on a reasonable request.
