# Supplementary material for: Impact of dengue fever on depression, anxiety, and stress symptoms in Esmeraldas Province, Ecuador: a prospective cohort study
Source: Trop Med Health. 2024 Sep 27;52:63. doi: 10.1186/s41182-024-00625-0 (PMC11428912; doi:10.1186/s41182-024-00625-0)
Supplement: Supplementary file 1 — Supplementary Material 1. [file 41182_2024_625_MOESM1_ESM.pdf]

S1 File Supporting Information

Dengue Esmeraldas Data

ID card number

____

Phone

____

Code

____

Date of birth

____
yyyy-mm-dd

Recruitment date: date on which you recruit the participant

____
yyyy-mm-dd

Health Center attended (where I pick you up)

San Rafael

La Concordia

Other

Ethnic group

Afro Mixed

White

Mulatto

Indigenous

Other

Date of diagnosis of Dengue

____

yyyy-mm-dd

Diagnostic mode

NS1

IGM

Clinical

Have you been diagnosed with Covid?

yes

no

How long ago did you have Covid?

less than 3

months from 3 to 6 months

more than six months

I have not had Covid

Clinical Background

Diabetes

Chikungunya

Heart failure Hypothyroidism

Rheumatoid arthritis or chronic joint disease (does not include osteoarthritis)

Other

Family history

depression

rheumatoid arthritis

none of the two

Alcohol consumption

Occasional

Problematic

Addiction

Does not consume

Tobacco use

Yes at least one daily

No

Education

Elementary

School High

School College

None

Approximate household income

less than $90

between $91 and $400

between 401 and 700

over 701

Occupation of head of household

Professional (working in their field of training or managerial position)

Semi-professional (middle management, oficinists)

Small business owner, agriculture, fishing Laborer   or raftsman with trade (plumber, electrician)
Assistant laborer

Untrained laborer

Unemployed

Life events

Death of a close family member in the last six months

Loss of employment in the last six months

Other adverse events in the last six months (robbery, violence...)

None

Housing

Cane-adobe cement

Mixed

S2 File Supporting Information

Dengue and depression DASS-21

Code

____

I had a hard time releasing the tension

0

1

2

3

I realized that my mouth was dry

0

1

2

3

I could not feel any positive feelings

0

1

2

3

It became difficult to breathe

0

1

2

3

I found it difficult to take the initiative to do things.

0

1

2

3

I overreacted in certain situations

0

1

2

3

I felt my hands trembling

0

1

2

3

I felt that I was expending a great deal of energy.

0

1

2

3

I was worried about situations in which I might panic or make a fool of myself.

0

1

2

3

I felt that there was nothing to look forward to

0

1

2

3

I have felt uneasy

0

1

2

3

I found it difficult to relax

0

1

2

3

I felt sad and depressed

0

1

2

3

I did not tolerate anything that did not allow me to continue with what I was doing.

0

1

2

3

I felt I was at the point of panic (losing control).

0

1

2

3

I couldn't get excited about anything

0

1

2

3

I felt I was worth very little as a person

0

1

2

3

I have tended to feel angry easily.

0

1

2

3

I felt my heart beating even though I had not made any physical effort.

0

1

2

3

I was afraid for no reason

0

1

2

3

I felt that life had no meaning

0

1

2

3

S3 file Supporting Information

The Cambridge Neuropsychological Test Automated Battery (CANTAB) used to computerized cognitive assessment, since 1980, it has 25 different tests which assess different cognitive domains. We conducted our research using two tests DMS (Delayed matched to sample) and SWM.

Delayed matched to sample (DMS)

This test assesses visual recognition memory; participants must recognize and exactly choose the model figure, that was presented at the top of the screen, among four similar figures that are presented at the bottom of the screen, in some trials the model figure does not disappear and in other trials the figure disappears and the similar options at the bottom take different time to appear. Several results can be obtained, the core ones being median correct latency, percent correct, probability of error given error, in general high scores show worse performance.

A video of how the test is done can be seen at the following link:

https://www.cambridgecognition.com/cantab/cognitive-tests/memory/delayed-matching-to-sample-dms/

Spatial working memory (SWM)

This test assesses strategy and working memory; participants must search for blue symbols hidden under colored squares, but they cannot search the site again they already found a blue symbol. Several results can be obtained, the core ones being strategy and the total times that a participant revisits a squared where a symbol was already founded, in general lower scores show better performance.

A video of how the test is done can be seen at the following link:

https://www.cambridgecognition.com/cantab/cognitive-tests/executive-function/spatial-executive-function-swm/
